# Development and In Vivo Evaluation of Multidrug Ultradeformable Vesicles for the Treatment of Skin Inflammation

**DOI:** 10.3390/pharmaceutics11120644

**Published:** 2019-12-03

**Authors:** Roberto Molinaro, Agnese Gagliardi, Antonia Mancuso, Donato Cosco, Mahmoud E. Soliman, Luca Casettari, Donatella Paolino

**Affiliations:** 1 Department of Biomolecular Sciences, University of Urbino Carlo Bo, 61029 Urbino (PU), Italy; roberto.molinaro@uniurb.it; 2 Department of Experimental and Clinical Medicine, University Magna Graecia of Catanzaro, Loc. Germaneto I-88100 Catanzaro, Italy; gagliardi@unicz.it; 3 Health Sciences Department, University Magna Graecia of Catanzaro, Loc. Germaneto I-88100 Catanzaro, Italy; antonia.mancuso@unicz.it (A.M.); donatocosco@unicz.it (D.C.); 4 Department of Pharmaceutics and Industrial Pharmacy, Faculty of Pharmacy, Ain Shams University, Cairo 11566, Egypt; mahmoud.e.soliman@pharma.asu.edu.eg

**Keywords:** ultradermable vesicles, Tween^®^ 80, sodium deoxycholate, naproxen sodium, idebenone, inflammation, transdermal drug delivery

## Abstract

The aim of this work was to evaluate the effect of two chemically different edge activators, i.e., Tween^®^ 80 and sodium deoxycholate, on (i) the physical, mechanical, and biological properties of ultradeformable vesicles, and (ii) the administration of naproxen sodium-loaded multidrug ultradeformable vesicles for the transdermal route in order to obtain therapeutically meaningful drug concentrations in the target tissues and to potentiate its anti-inflammatory effect by association with the antioxidant drug idebenone. The results obtained in this investigation highlighted a synergistic action between naproxen and idebenone in the treatment of inflammatory disease with a more pronounced anti-inflammatory effect in multidrug ultradeformable vesicles compared to the commercial formulation of Naprosyn^®^ gel. Systems made up of Tween^®^ 80 appeared to be the most suitable in terms of percutaneous permeation and anti-inflammatory activity due to the greater deformability of these vesicles compared to multidrug ultradeformable vesicles with sodium deoxycholate. Our findings are very encouraging and suggest the use of these carriers in the topical treatment of inflammatory diseases.

## 1. Introduction

Over the last decades, the possibility of using the transdermal route as the preferential way to administrate anti-inflammatory drugs for the treatment of local or systemic inflammatory states has been seriously considered [1]. However, conventional formulations, as ointments, creams, or gels, are subject to many disadvantages, linked especially to the barrier function of skin, in particular the stratum corneum. This latter, in fact, due to its particular composition (stratum corneum consists of an extracellular lipid matrix made up of ceramides, cholesterol, long chain fatty acids at a defined molar ratio), hinders the passage of substances, especially hydrophilic compounds, by reducing the efficacious amount of drug that can perform a pharmacological action [2]. Moreover, for conventional topical formulations, the release of drugs from the vehicle and its subsequent passage through the skin is strongly influenced by the physico-chemical properties of the medicament itself [3]. As a matter of fact, the best results in terms of skin permeation have been achieved by using amphiphilic drugs.

Most of these limitations have been overcome by transdermal drug delivery systems [4]. Among these, vesicular systems belong to the most interesting methods for transdermal delivery of active substances. The interest in these kind of carriere results from their ability to act as penetration enhancers for the delivery of a payload across the skin [5]. There are two classes of vesicular carriers, rigid and ultradeformable ones. The first ones include liposomes and niosomes and are characterized by a rigid, non-deformable membrane, which hampers them in reaching the deeper layers of the skin, thus remaining confined to the stratum corneum [6,7]. Conversely, elastic vesicles, such as ultradeformable vesicles (e.g., transfersomes^®^) or ethosomes^®^, consist of an ultra-flexible membrane that permits them to squeeze through narrow skin pores and reach subcutaneous tissue [8]. The driving force that allows the carrier to penetrate through the skin is represented by a transepidermal water-activity gradient [9], due to the different hydration status of the skin surface (approximately 20% water) compared to the viable epidermis (close to 100%). Due to this gradient, ultradeformable carriers move from a dry environment to one with a higher content of water. This xerophobia [10] is strictly related to the composition of the carrier, made of polar lipids and an edge activator, i.e., a surfactant that destabilizes the membrane package making it elastic, thus allowing the carrier to deliver more than 50% of the drug through the skin barrier [11]; it is a significant value considering that the topical bioavailability of the most commercialized formulations is very low (around 1%–5% of the applied amount). Added to this, their ability of obtaining a topical or a systemic effect [12] and their physical stability [13] represent important parameters in the context of their pharmaceutical development.

In 2007, the Transfersome^®^ formulation Diractin^®^ containing the non-steroidal anti-inflammatory drug (NSAID) ketoprofen, was approved by the Swiss regulatory agency (SwissMedic) for the management of osteoarthritis. [14].

In recent years several anti-inflammatory drugs such as baicalin [15], methotrexate [16], naringenin [17], resveratrol [18,19], and naproxen sodium [20] have been encapsulated in ultradeformable liposomes in order to reduce skin inflammation. The current study was focused on the co-encapsulation of two active compounds, naproxen sodium and idebenone, within ultradeformable liposomes, made up of two different edge activators, i.e., Tween^®^ 80 (T80), a hydrophilic non-ionic surfactant, and sodium deoxycholate (SDC), a steroid-like anionic surfactant (Formulations 1 and 2, respectively) in order to obtain a synergistic action and thus to potentiate the anti-inflammatory effect. The entrapment efficiencies of idebenone (IDE) at 5% and 10% *w*/*v* (Formulations A and B, respectively) and naproxen sodium (NS) at 0.4% and 1% *w*/*v* (Formulations C and D, respectively), either alone or in combination (Formulation E) were investigated for their potential application in the treatment of inflammation. Moreover, the percutaneous permeation in vitro through human stratum corneum and viable epidermis membranes was evaluated and then their permeation profiles were compared with the ones of the NS-containing commercial gel. Finally, the anti-inflammatory efficacy, the prolonged release properties of UVs, and their tolerability were investigated in human volunteers.

The rationale of combining NS and IDE was derived from evidence that, during the developing of an inflammatory state, the production of free radicals aggravates the inflammation due to the damage they cause to cell and mitochondrial membranes [21]. IDE (2,3-dimethoxy-5-methyl-6-(10-hydroxydecyl)-1,4-benzoquinone) is a lipid-soluble antioxidant, able to scavenge the reactive oxygen species (ROS) [22].

## 2. Materials and Methods

### 2.1. Materials

Enriched soy phosphatidylcholine (Phospholipon^®^ 90G, P90G) was supplied by Lipoid GmbH (Ludwigshafen, Germany), SDC, T80, NS, IDE and metilnicotinate were purchased by Sigma-Aldrich (St. Louis, MO, USA); ethanol (95°) was acquired from Carlo Erba (Milan, Italy). Naprosyn^®^ gel 10% is a commercial formulation of NS produced by Recordati S.p.A. (Innova Pharma, Milan, Italy). In order to evaluate the release profiles, cellulose dialysis membranes with cutoff of 10,000 Daltons were purchased from Prodotti Gianni S.p.a (Milan, Italy). All materials used were high purity and did not require further purification procedures prior to use.

### 2.2. Ultradeformable Vesicles Preparation

The UVs were prepared according to the thin layer evaporation method. Briefly, PL 90G^®^ and the edge activators, i.e., SDC or T80 (100 mg) were dissolved in 3 mL of ethanol in a Pyrex^®^ glass vial. The organic solvent was removed by a Rotavapor^®^ 210 (Büchi Italia, Milan, Italy) under a nitrogen flux and then by overnight storage at 30 °C in a Büchi T51 glass drying-oven under vacuum. In Table 1, the components that constitute the ultradeformable carriers and their ratios are presented. The obtained film was stored at 4 °C for 12 h and subsequently hydrated with 6 mL of water/ethanol (93:7 *w*/*w*) under continuous stirring for 15 min. The prepared multi-lamellar vesicles had high values of mean sizes (about 450 nm) and a polydispersity index (greater than 0.3).

In order to obtain unilamellar vesicles suitable for skin delivery, the formulations were extruded through polycarbonate filters with pores of 200 nm in diameter by means of a Lipex Extruder^TM^ (Vancouver, BC, Canada). Subsequently, the sample was left for 2 h at 60 °C and then for 24 h at 4 °C for stabilization. For the preparation of multidrug UVs, the two active compounds were added during the preparation phases according to their solubility. IDE was added to the lipophilic phase before the formation of the thin film, while NS was added to the aqueous solution.

### 2.3. Physicochemical Characterization

The mean sizes and polydispersity index of the different formulations were determined by dynamic light scattering (DLS) analysis. The instrument used was a Nano Zetamaster ZS (Malvern Instruments, Worcestershire, UK), equipped with a laser diode with a rated output of 4.5 mV, set at a wavelength of 670 nm and a backscattering angle of 173°. To avoid multi-scattering phenomena, samples were diluted with an isotonic solution of distilled water previously filtered through a polypropylene membrane (average pore size of 0.22 μm) (Whatman Inc., Clifton, NJ, USA). The different suspensions were placed in a quartz cuvette. For each sample 10 different measurements were performed at room temperature. The Zetasizer Nano ZS was also used for Z-potential determination by applying a Smoluchowsky constant F (Ka) of 1.5 to calculate the zeta-potential value as a function of the electrophoretic mobility of the nanoparticles. The various measurements were carried out in triplicate on three different batches (10 determinations for each batch). Results were expressed as the mean of three different experiments ± standard deviation [23].

TurbiscaLab^®^ Expert (Formulaction, France) was used to evaluate physical stability of the various formulations. The obtained curves provide the transmitted and backscattered light flux in percentage relative to standards (suspension of monodisperse spheres and silicone oil) as a function of the sample height (in mm). These profiles build up a macroscopic fingerprint of the sample at a given time. Formulations were compared to each other by calculating the stability kinetics through the evaluation of the Turbiscan Stability Index (TSI) (the lower the TSI, the higher the formulation stability) [24].

### 2.4. Deformability Index (DI) Evaluation

The main feature of multidrug UVs is their ability to deform and pass intact through the skin. Vesicle deformability was assessed by extrusion assay [25]. Briefly, each vesicular dispersion was extruded at a constant pressure of 20 bar through polycarbonate filters (Nucleopore Polycarbonate) with defined holes (50 nm) by using the extruder Lipex Extruder^TM^. The deformability of the vesicles was expressed in terms of deformability index (DI), according to Equation (1):

DI = *J* × (*d*_0_/*p*)*^n^* × (1/(*d*_1_ − *d*_0_)) (1)

where *J* is the fraction of the suspension collected after extrusion (a value that varies between 0 and 1), *d*_0_ and *d*_1_ are the average size before and after the extrusion, *p* is the diameter of the pores of the polycarbonate membrane, and *n* is an amplification factor.

### 2.5. Evaluation of Encapsulation Efficiency (EE)

The amount of active ingredients encapsulated in vesicular carriers was assessed by the ultracentrifugation method [26]. In particular carriers containing the two drugs, alone or in combination, were centrifuged at 95,000 rpm for 1 h at 4 °C using the Beckman Avanti™ 30 Centrifuge (Beckman Inc. Conter, Fullertan, CA, USA), with a fixed-angle rotor (TLA-100.4). The sediment obtained, consisting of precipitated vesicles, was separated from the supernatant. The amount of encapsulated drugs was evaluated by HPLC analysis at the wavelengths of the two active ingredients and indirectly calculated as a difference between the amount of drug added during the preparation and the amount of the unentrapped drug present in the supernatant. The actual amount of the drug present in the supernatant was determined using the following Equation (2):

EE = ((*D*_t_ − *D*_u_)/*D*_t_) × 100 (2)

where *D*_t_ is the total amount of the drug used for carrier preparation and *D*_u_ is the amount of untrapped drug.

### 2.6. HPLC Determination of NS and IDE

The amounts of the two encapsulated and released active compounds from UVs were determined by using a Varian HPLC chromatographic system (Varian Inc., Palo Alto, CA, USA), equipped with an injector loop CSL20 Cheminert of 20 µL. The determination of the two active ingredients was conducted using a reversed-phase chromatography column Pursuit RP18 (150 × 4.6 mm, 5 μm i.d., Varian Inc., Yarnton, UK) at room temperature. Data were acquired and processed with a Galaxie chromatography manager software (Varian Inc., Palo Alto, CA, USA). IDE and NS were detected at a wavelength of 282 and 331 nm, respectively. Chromatographic separation was performed by using as mobile phase a water/acetonitrile mixture, acidified with 0.01% trifluoroacetic acid (TFA), with a gradient elution from 80:20 to 20:80 in 20 min. The flow rate was set at 1 mL/min. To quantify the active ingredients, a calibration line was constructed bringing in a system of axes for the concentration of known solutions of drug with respect to the relative AUC. The following NS (Equation (3)) and IDE (Equation (4)) calibration curves were used:

AUC = 3 × 10^−6^*x* + 0.6904 (3)

AUC = 9 × 10^−8^*x* + 0.2461 (4)

where *x* is the drug concentration (µg/mL) and AUC the area under the curve (mAu × min). The calibration lines are linear in the concentration range between 0.1 and 20 µg/mL.

### 2.7. Ex Vivo Skin Penetration and Permeation Studies

#### 2.7.1. Preparation of Stratum Corneum Epidermis (SCE) Membranes

Evaluation of skin permeability in vitro of UVs containing IDE and NS was carried out using samples of human skin made only by the stratum corneum and epidermis (SCE). Samples of human skin, used in in vitro permeation experiments, were obtained by reductive plastic surgery, performed on adult male subjects (29 ± 4 years), in the abdominal region (approved by Ain Shams University – Cairo EGYPT: ENREC-ASU-2019-97, 11 November 2019. The evaluation of the skin permeability was performed using skin samples consisting only of SCE, as the use of whole skin in in vitro percutaneous absorption experiments of lipophilic substances can give unreliable results because the dermis can behave as an additional barrier to permeation [8]. The separation of dermis from SCE was carried out according to the procedure described by Kligman and Chistophers (1963) [27]. Briefly, the skin samples, after careful removal of subcutaneous fat, were immersed in distilled water at 60 ± 1 °C for 2 min after which the stratum corneum and epidermis were gently removed from the underlying dermis with help of a scalpel. SCE membranes thus obtained were dried in a desiccator (25% RH), wrapped in aluminum foils and stored at 4 ± 1 °C until use. This preservation technique allows the characteristics of permeability of the SCE samples to be maintained unchanged for at least 9 months.

To assess the integrity of the barrier properties of the samples of SCE, these samples were subjected to preliminary experiments of in vitro permeation using tritiated water as a permeating agent. The value of the permeability coefficient (Kp) for tritiated water determined for these samples was 1.7 ± 0.3 × 10^-3^ cm/h and was in good agreement with that reported by other authors [28] for human SCE samples whose barrier properties were perfectly intact.

#### 2.7.2. In Vitro Evaluation of the Permeation of Compounds from Ultradeformable Carriers

The in vitro permeation experiments were performed in non-occlusive conditions by using Franz diffusion vertical cells (LGA, Berkeley, CA, USA) [29]. Each cell consists of a donor chamber, in which is placed the formulation and a second chamber, the receptor, containing the receptor solution, consisting of a mixture of water/ethanol (60:40 *v*/*v*) which is continuously stirred with a small magnetic bar and thermostated at 37 ± 1 °C throughout the experiment to reach the physiological skin temperature (i.e., 32 ± 1 °C). SCE membranes were placed between the donor and the receptor, positioning them so that the SC was directed toward the donor [30]. The skin surface area available for permeation was 0.75 cm^2^ while the volume of the acceptor compartment below the membrane was 4.5 mL [31].

To evaluate the effect on skin permeation of UVs, the commercial formulation Naprosyn^®^ gel 10% was used as reference. An amount of 200 µL of vesicular formulation (equivalent to 2 mg of NS and 0.33 mg of IDE) or the equivalent amount of commercial formulation to be tested was applied on the skin surface and the amount was sufficient to maintain steady state conditions. At fixed time intervals (1, 2, 4, 6 and 8 h) samples were collected (about 360 µL) from the receptor solution and analyzed by HPLC for drug content. The withdrawn volume was replaced with fresh medium and a correction for dilution was carried out. Six different permeation experiments were carried out for each formulation and the results are expressed as the mean value ± the standard deviation.

### 2.8. Evaluation of Release Profiles of Drugs from Vesicular Carriers

The rate of release of drug from the formulations was evaluated using cellulose membranes with a cut-off of 10,000 Daltons. The membranes were hydrated before starting the experiment in distilled water for 40 min at room temperature to remove the sodium azide present and then were filled with 1 mL of different formulations. Subsequently, the membranes containing the formulations were placed in borosilicate glass beaker containing 200 mL of receptor phase consisting of a mixture of water/ethanol (60:40 *v*/*v*). Then 1 mL of solution was taken from each beaker at different times (30 min, 1, 2, 4, 6, 8, 12 and 24 h) and was replaced with an equal volume of receptor solution. The samples were analyzed by HPLC at the wavelengths of the two drugs.

### 2.9. In Vivo Tolerability on Human Volunteers

According to the work of Paolino and coworkers [32], the in vivo applicability of vesicular carriers containing two active ingredients was evaluated by using an in vivo non-invasive method. It consists of spectrophotometry of reflectance, which uses the reflectance spectrophotometer SP60 (X-Rite Incorporated, Grandville, Michigan, USA), able to detect any changes in skin color due to the variation of the two physiologically present chromophores in human skin, i.e., melanin and hemoglobin. We enrolled healthy volunteers (*n* = 12), who provided their written consent, previously informed about the aim and procedures of the study. Subjects had not taken any medication for at least 1 week and rested at room conditions (22 ± 2 °C and 40%–50% r. h.) for 30 min before the experiments.

The experimental protocol followed for the in vivo experiments was the following: six sites (three per arm) upon the medial part of both forearms were demarcated in twelve healthy volunteers and on everyone a first measurement with the reflectance spectrophotometer before application (baseline) was carried out. The sites were randomly defined using a circular template (1 cm^2^). The distance between sites was at least 2 cm in order to avoid any possible interference. After the baseline detection, the empty formulations 1 and 2 were applied on two of the three sites using Hill Top chambers (Hill Top Re-search, Inc. Cincinnati, Miamiville, OH, USA). On the third site (for each arm), saline solution was applied as control. Before reflectance spectrophotometric readings, the chambers were removed and the skin surface was gently washed with water to remove the applied formulation and the skin was allowed to dry for 15 min. The possible induced erythema (EI) was monitored at 6, 24, and 48 h and was calculated according to the following equation (Equation (5)).

(5) $$I.E.=\;100\left[log\frac{1}{R560}+1.5\left(log\frac{1}{R540}+log\frac{1}{R580}\right)-2\left(log\frac{1}{R510}+log\frac{1}{R610}\right)\right]$$

where 1/*R* is the inverse reflectance at a specific wavelength, in particular 540, 560, and 580 represent the absorption peaks of hemoglobin, while 510 and 610 are those of melanin.

The baseline values of the IE, determined for each site prior to treatment with the formulation under consideration, are subtracted from the values of IE calculated as a function of time for the same site, thus obtaining ΔEI. This latter is an important parameter in the evaluation of the erythema, because the higher the ΔEI values the greater the intensity and duration of erythema and, therefore, the skin toxicity of the tested formulations.

### 2.10. In Vivo Anti-Inflammatory Activity

The in vivo efficacy of UVs containing IDE and NS, either alone or in combination, was compared to saline solution and commercial Naprosyn^®^ gel 10%. In particular, we evaluated their ability to reduce the chemically induced erythema through the following procedure: eight sites on the ventral surface of each forearm (a total of eight subjects) were randomly defined using a circular template (1 cm^2^ in diameter) and demarcated with permanent ink. The distance between sites was at least 2 cm in order to avoid any possible interference, all the sites were treated with 200 µL of an aqueous methyl nicotinate solution (0.2% *w*/*v*) for 15 min using Hill Top Chambers. After the topical treatment with methyl nicotinate, we removed the Hill Top Chambers and gently washed the skin surface with water to remove the applied formulation. Then, we applied 200 µL of the various formulations (formulation 1B: T80-containing 10% *w*/*w* IDE-loaded UVs; formulation 1D: T80-containing 1% *w*/*v* NS-loaded UVs; formulation 1E: T80-containing 10% *w*/*w* IDE-, 1% *w*/*v* NS-loaded UVs, formulation 2B: SDC-containing 10% *w*/*w* IDE-loaded UVs; formulation 2D: SDC-containing 1% *w*/*v* NS-loaded UVs; formulation 2E: SDC-containing 10% *w*/*w* IDE-, 1% *w*/*v* NS-loaded UVs, saline and Naprosyn^®^ gel). The induced erythema was monitored by mean of reflectance spectrophotometric readings until its disappearance. In addition, we evaluated in vivo the controlled release properties of our formulations using a pre-treatment approach. Briefly, we randomly defined eight sites on the ventral surface of each forearm using a circular template (1 cm^2^). Sites were demarcated at a distance of at least 2 cm from one to the other with permanent ink. Then, 200 µL of the formulations above reported were used to treat sites (in double) for different time points (1, 3, and 5 h) using Hill Top chambers (1 cm^2^). Following the pretreatment period, we removed the chambers, washed the skin surface to remove the formulations, and applied a Hill Top Chamber containing 100 µl of methyl nicotinate (0.2% *w*/*v*) for 15 min. At the end of the treatment, we monitored the induced erythema through reflectance spectrophotometry until the complete disappearance of erythema as previously described [32].

### 2.11. Statistical Analysis

Statistical analysis of the various experimental results was performed by using one-way ANOVA. A posteriori Bonferroni t-test was carried out to check the ANOVA test. A *p* value <0.05 was considered statistically significant. All the values are reported as the average ± standard deviation.

## 3. Results and Discussion

### 3.1. Physicochemical Characterization of UVs

DLS analysis evidenced that, after extrusion, all the formulations were characterized by a mean size lower than 150 nm and low values of polydispersity index (<0.13), which were not affected by the encapsulation of two active compounds (Table 2). We observed, instead, a difference in zeta-potential values, probably due to the different chemical nature of the two edge activators in terms of both steric hindrance and net charge, especially for SDC. Indeed, formulations containing SDC had zeta potential values (between −33 and −40 mV) lower than those containing T80 (between −24 and −33 mV) (Table 2).

It is interesting to note that the simultaneous presence of both drugs in Formulations 1E and 2E resulted in a reduction of zeta potential values of at least 10 mV compared to the empty formulations (−20.3 and −27.6 mV, respectively).

**Figure 1:**
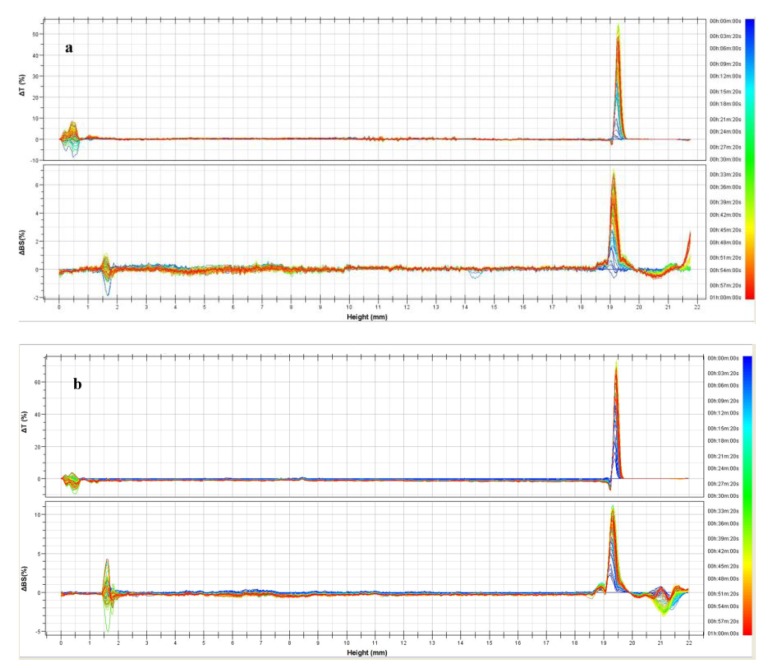
Transmittance (ΔT) and Backscattering (BS) profiles evaluated by Turbiscan Lab^®^ Expert: (**a**) Formulation 1; (**b**) Formulation 2.

Next, we used Turbiscan Lab^®^ Expert to detect the presence of migration phenomena within the samples and therefore to evaluate their physical stability. For the empty formulations (Formulations 1 and 2), the variation of delta backscattering is always lower than ±5% (Figure 1), thus indicating their long-term stability (until six months) [33,34].

**Figure 2:**
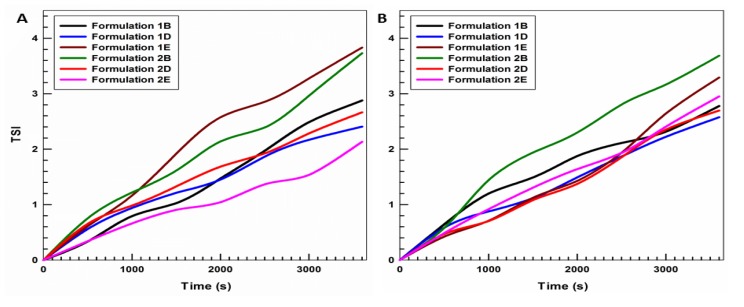
Turbiscan Stability Index (TSI) of ultradeformable formulations containing NS and IDE. Values are the mean of three different experiments ± standard deviation. *T* = 20 °C (panel **A**), *T* = 37 °C (panel **B**).

The addition of NS and IDE, either alone or in combination, did not induce any change in the stability profiles, maintaining a delta backscattering variation less than ±1% (data not showed), as also revealed by the evaluation of the stability index (TSI) (Figure 2). As a matter of fact, the encapsulation of different amounts of active principle did not result in significant variations of the stability index compared to the empty carriers at both 20 and 37 °C (Figure 2A,B, respectively). This phenomenon confirms the ability of UVs to effectively retain the payload without inducing destabilization phenomena.

Next, we evaluated the efficiency of drug encapsulation by the two formulations. Table 3 shows a high entrapment efficiency of IDE for both UVs. However, the presence of SDC as edge activator reduces the encapsulation of IDE, probably due to the bulky structure of this surfactant; on the contrary, formulations containing T80 showed the highest encapsulation efficiency of IDE (73% and 82% for Formulations 1A and 1B compared to 51% and 72% for the Formulations 2A and 2B, respectively). As regard NS, Formulation 1 showed the best values of entrapment efficiency. T80, in fact, due to its non-ionic nature, attenuates the repulsion forces between P90G and NS favoring its encapsulation. Conversely, the anionic carboxylic group of SDC increases the repulsion forces, thus decreasing the amount of hydrophilic drug encapsulated in the core of the nanovesicles. In both cases, the greater the amount of drug initially added during the preparation the higher was the entrapment efficiency; in particular, at the highest drug concentration used (1% *w*/*v*), the percentages of entrapment efficiency are 30.1% and 20% for formulations 1D and 2D, respectively (Table 3). When the two drugs are co-encapsulated, this charge-shielding effect is even more evident. As can be seen in Table 3, the presence of IDE favors the subsequent encapsulation of NS, in the presence of both SDC (29%) and T80 (40%), while the percentage of IDE is not significantly affected.

The different structure of the two edge activators affected also the deformability of UVs. The main feature of the UVs is the elasticity of their membrane, that allows them to cross intact the skin and the stratum corneum due to the transepidermal water-activity gradient [35]. Extrusion of vesicles through 50 nm pore-polycarbonate filters was performed to evaluate carrier deformability properties. In Table 4 we report the mean sizes, before and after the extrusion, and the relative deformability indices of both Formulations 1 and 2 and a liposomal formulation, taken as control, consisting of P90G and cholesterol (70:30 molar ratio). It is possible to note that both edge activators increased membrane deformability (DI = 109.73 and 37.65 for Formulations 1 and 2) with respect to liposomal formulation (DI = 17.75) and evidenced a significantly higher deformability induced by T80 compared to SDC. This feature could be related to the linear, non-bulky structure of T80. Conversely, SDC, having a steroid-like structure, similar to that of cholesterol, tends to compact the bilayer thus reducing its flexibility.

The presence of the payload had a different effect on the UVs’ deformability. IDE positively affected the elasticity of the carrier, and this is probably due to its intercalation in the bilayer that causes a decrease in lamellar phase stability with an increase of deformability. The addition of the hydrophilic NS to the formulation, instead, caused a reduction, although not significant, of membrane elasticity compared to that of empty formulations (DI = 92.12 and 87.17 for Formulations 1C and 1D, respectively and 36.31 and 35.30 for Formulations 2C and 2D, respectively). The simultaneous presence of both drugs conferred to both formulations deformability indices slightly lower than that of the formulations at 10% *w*/*v* of IDE (DI = 172.56 and 76.08 for Formulations 1E and 2E, respectively).

**Figure 3:**
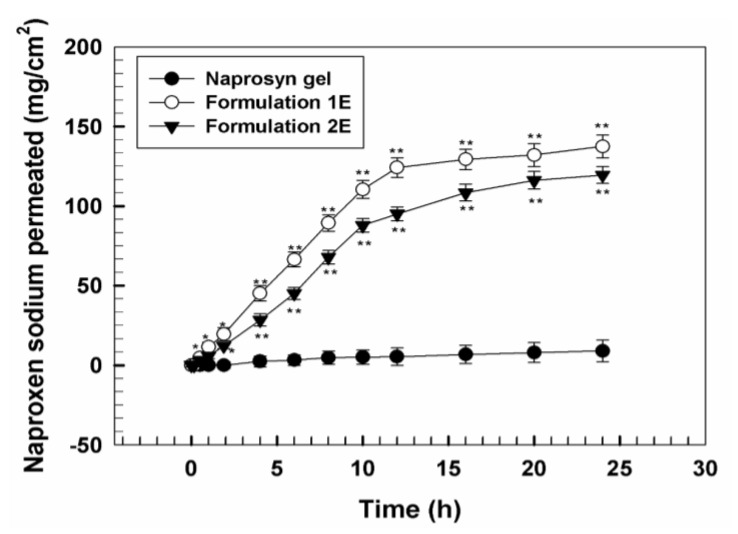
In vitro percutaneous permeation of multidrug ultradeformable vesicles (UVs) containing as edge activator Tween^®^ 80 (formulation 1E) or sodium deoxycholate (formulation 2E) through human SCE membranes. An NS commercial gel (Naprosyn^®^ 10%) was used as control. Values represent the mean of three different experiments ± standard deviation. * *p* < 0.05, ** *p* < 0.001 with respect to the control.

Figure 3 shows the percutaneous permeation profiles of UVs prepared with the two different edge activators with respect to commercial Naprosyn^®^ gel, computed as µg/cm^2^ of permeated NS and performed up to 24 h. Ultradeformable carriers were able to significantly (*p* < 0.001,) increase the percutaneous permeation of NS through human SCE membranes.

**Figure 4:**
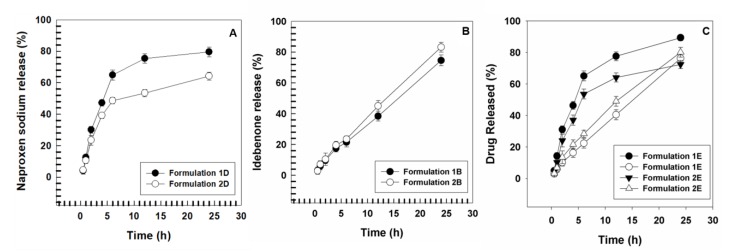
Release profiles of: (**A**) NS and (**B**) IDE from ultradeformable nanovesicles after encapsulation in single form (panel **A** and **B** respectively) and in association (panel **C**). The experiments were carried out at room temperature. Values represented the mean of three different experiments ± standard deviation. Filled symbols stand for NS; empty symbols stand for IDE.

In particular, profiles obtained showed that the amount of drug which permeated up to 24 h was 137.5 µg/cm^2^ when formulation 1E was used, while it was lower for formulation 2E (119.5 µg/cm^2^). For commercial gel it was very low (9.06 µg/cm^2^), with a lag time of about 2 h while, for UVs, the lag time was much shorter (about 15 min). This trend reflects the higher deformability of formulation 1E in comparison with formulation 2E (Figure 4).

Release profiles of IDE and NS, alone or in combination, from UVs were performed through cellulose membranes with cutoff of 10,000 Da for up to 24 h.

**Figure 5:**
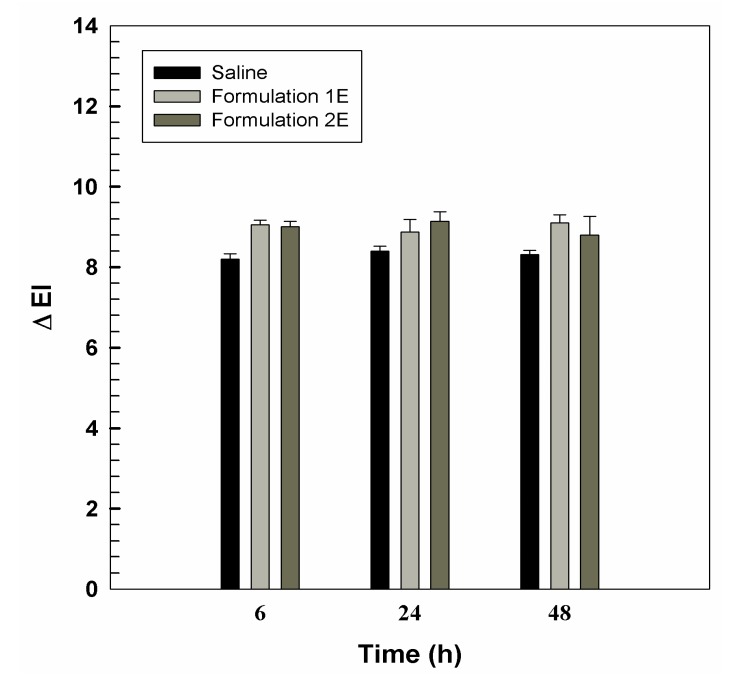
In vivo human skin tolerability of empty topical formulations 1 and 2, after 6, 24 or 48 h of treatment. Results are expressed as a mean value of ΔEI (*n* = 6) ± standard deviation.

Figure 4 shows the release profile of NS from formulations 1 D and 2 D. In both cases, the release is biphasic and is characterized by an early phase (up to 6 h) in which it is rapid and linear and a secondary one (up to 24 h) in which, instead, the release is constant and gradual, with a zero-order kinetic profile, typical of a drug-reservoir system. It is interesting to note that the edge activator affects the amount of drug that is released. In fact, as above mentioned, the presence of SDC confers rigidity to the bilayer, thus reducing the released amount of the encapsulated NS, that is around 48% after 6 h and 64% after 24 h for formulation 2D in comparison with 64% after 6 h and 79% after 24 h for formulation 1D. On the contrary, the release of IDE from formulations 1B and 2B was constant and linear throughout all the 24 h and the released drug amount was higher for SDC-containing formulation (about 83% after 24 h) with respect to the T80 one (about 74%) (Figure 5), thus validating previous reports from Jain and coworkers [36]. When the two drugs were co-encapsulated, the amount of NS released after 24 h was higher than in the formulation alone (about 89% and 72% after 24 h for formulations 1E and 2E, respectively), while no significant variation was observed for the IDE release profile from formulations 1E and 2E (about 76% and 80% after 24 h, respectively).

### 3.2. In Vivo Tolerability on Human Volunteers and Evaluation of Anti-Inflammatory Activity

An important parameter to evaluate before proposing a carrier for drug delivery is its in vivo skin tolerability. Here, we performed reflectance spectrophotometry in order to assess the eventual occurrence of skin erythema after the application of empty carriers. The results of skin tolerability are expressed as variation of the erythema index (ΔEI) (Figure 5). The skin tolerability of UVs was compared to that of a saline solution, taken as control. The experiments were carried out on healthy volunteers after 6, 12, 24, and 48 h from the application. The obtained data revealed as ΔEI for both UVs did not vary significantly with respect to control, thus indicating the tolerability of the carriers.

**Figure 6:**
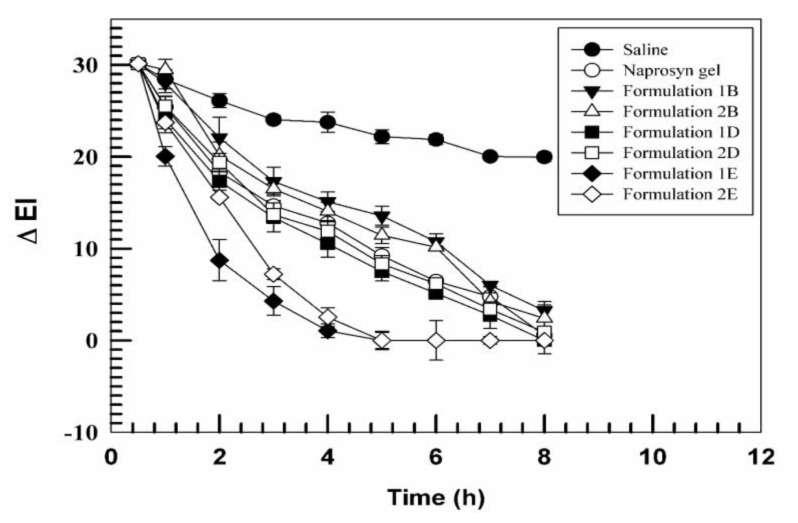
Variation of erythema index (ΔEI), chemically-induced by methyl nicotinate (0.2% *w*/*v*), after treatment with different formulations in comparison with a negative and a positive control (saline and Naprosyn^®^ gel, respectively) as function of time. Results are expressed as a mean value (six different volunteers) of the erythema index variation ± standard deviation as a function of the time.

Therefore, having ascertained the in vivo safety of formulations, the subsequent step was the evaluation in vivo of their anti-inflammatory activity with respect to commercial Naprosyn^®^ gel 10% in order to fully understand their therapeutic potential. As we previously reported [32], first we chemically induced the erythema by treating the skin with a 0.2% *w*/*v* methyl nicotinate solution, and then we treated those sites with our formulations. Figure 6 shows the ΔEI of various formulations as function of time. UVs were able to decrease the EI more rapidly than when the sites were treated with the commercial formulation Naprosyn^®^ gel. Specifically, formulations containing the anti-inflammatory drug alone (formulations 1D and 2D) had comparable efficacy, if not better in the case of formulation 1D, with respect to that of the commercial gel. The administration of formulations 1E and 2E, instead, induced a significant reduction of ΔEI, value that after 5 h was zero. The increased efficacy of the T80-containing formulations is to be found in the greater deformability of the carrier when this type of edge activator is used, they tend to reach more quickly and more effectively the underlying dermis, by stimulating the release of encapsulated active ingredients.

To evaluate the sustained effect on the release of UVs, we performed a pre-treatment assay. The sites were pretreated with the various formulations containing IDE or NS, either together or separately, for different time points (1 h, 3 h, and 5 h), as reported in the Methods section.

**Figure 7:**
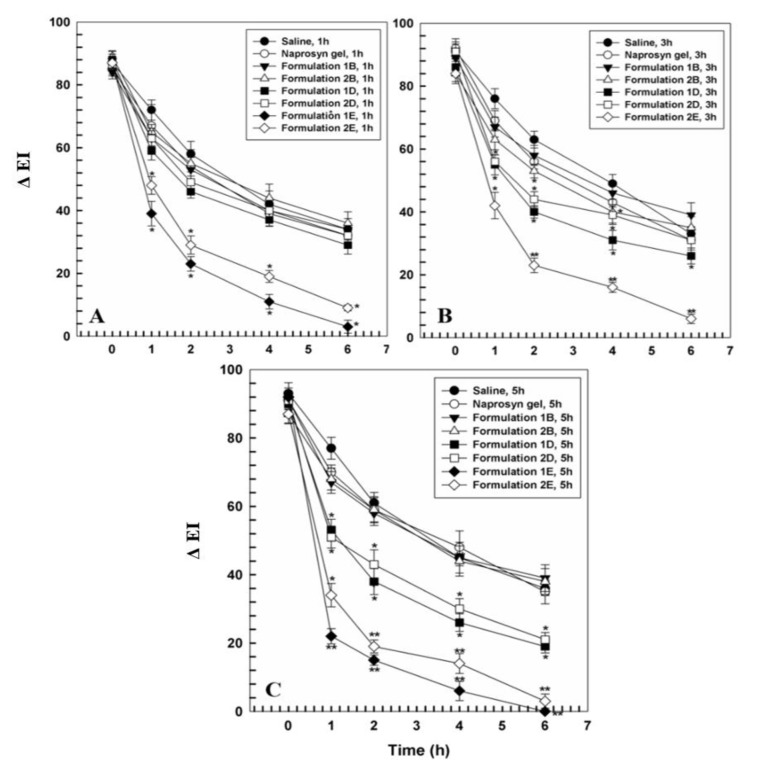
Variation of erythema index (ΔEI), chemically-induced by pretreatment with different formulations after treatment with a solution (200 µL) of methyl nicotinate (0.2% *w*/*v*) in comparison with a negative and a positive control (saline and Naprosyn^®^ gel, respectively) as function of time, 1 h (panel **A**), 3 h (panel **B**) and 5 h (panel **C**). Results are expressed as a mean value (six different volunteers) of the erythema index variation ± standard deviation as a function of the time. * *p* < 0.05, ** *p* < 0.001.

In Figure 7 it is possible to observe how all the pre-treated sites formulations containing the anti-inflammatory drug alone (formulation 1D and 2D) were able to reduce significantly the onset of erythema after 3h and 5h of pretreatment, showing that UVs are able to improve the amount of permeated anti-inflammatory drug through the skin. Indeed, the combination of the two drugs led to a reduction of the onset of erythema after all the pretreatment times, with respect to Naprosyn gel and the NS formulations, thus confirming the synergistic effect of IDE and NS. In particular, the erythema onset, in case of multidrug UVs, followed the order: 5 h < 3 h < 1 h, showing that T80 was more efficacious as compared to the SDC formulations.

## 4. Conclusions

UVs realized in this work showed favorable physicochemical characteristics for their transdermal application. In particular, they had mean sizes lower than 200 nm, with a negative zeta potential (−20 mV). Turbiscan^®^ analysis revealed a physical stability for all the formulations. Moreover, these delivery systems are able to deliver drugs through the skin more efficiently than commercial Naprosyn^®^ gel and to release gradually the encapsulated active ingredients over 24 h, thus working as depot systems. T80^®^-containing UVs showed the best features in term of permeation and release profiles and in vivo anti-inflammatory activity; this is related to the higher deformability of these vesicles with respect to the sodium deoxycholate-containing ones; this allowed them to efficaciously reach deeper layers of the skin. This ability, combined with the synergistic action of NS and IDE on the inflammatory process, led UVs to have an anti-inflammatory activity, measured as variation of the erythema index, more pronounced than commercial gel.

These findings are very encouraging and suggest the use of these carriers as potential therapeutic strategy for the treatment of localized inflammatory states.

## Figures and Tables

**Table 1:** Quali-quantitative composition of various vesicular formulations.

Formulation	P90G ^1^ (% *w*/*w*)	SDC ^2^ (% *w*/*w*)	T80 ^3^ (% *w*/*w*)	IDE ^4^ (% *w*/*w*)	NS ^5^ (% *w*/*v*)
1	85	-	15		-
1A	80	-	15	5	-
1 B	75	-	15	10	-
1C	85	-	15	-	0.4
1D	85	-	15	-	1
1E	75	-	15	10	1
2	90	10	-	-	-
2A	85	10	-	5	-
2B	80	10	-	10	-
2C	90	10	-	-	0.4
2D	90	10	-	-	1
2E	80	10	-	10	1

^1^ Phospholipon^®^ 90G; ^2^ Sodium deoxycholate; ^3^ Tween^®^ 80; ^4^ Idebenone; ^5^ Naproxen sodium.

**Table 2:** Physicochemical properties of empty ultradeformable nanovesicles (1 and 2), IDE-loaded (1A, 1B, 2A, 2B), NS-loaded (1C, 1D, 2C, 2D) and multidrug ultradeformable vesicles (1E, 2E).

Sample	Mean Sizes (nm)	Polydispersity Index	Zeta Potential (mV)
1	136 ± 3	0.13 ± 0.01	−26 ± 2
1A	137 ± 2	0.10 ± 0.01	−33 ± 1
1B	134 ± 3	0.08 ± 0.03	−36 ± 2
1C	141 ± 3	0.09 ± 0.01	−24 ± 1
1D	131 ± 2	0.07 ± 0.01	−26 ± 1
1E	128 ± 3	0.09 ± 0.01	−20 ± 1
2	137 ± 2	0.11 ± 0.01	−37 ± 1
2A	144 ± 4	0.11 ± 0.01	−33 ± 1
2B	140 ± 3	0.08 ± 0.01	−40 ± 1
2C	137 ± 3	0.07 ± 0.02	−37 ± 1
2D	126 ± 2	0.12 ± 0.00	−39 ± 1
2E	132 ± 2	0.08 ± 0.01	−27 ± 1

**Table 3:** Entrapment efficiency (EE%) of NS and IDE.

Formulation	IDE EE% ± SD	NS EE% ± SD
1	-	-
1A	73 ± 1	-
1B	82 ± 1	-
1C	-	59 ± 1
1D	-	30 ± 1
1E	81 ± 1	40 ± 1
2	-	-
2A	51 ± 1	-
2B	72 ± 1	-
2C	-	44 ± 1
2D	-	20 ± 1
2E	72 ± 1	29 ± 1

**Table 4:** Deformability index (DI) of empty (1 and 2), IDE-loaded (1A, 1B, 2A, 2B), NS-loaded (1C, 1D, 2C, 2D) and multidrug ultradeformable vesicles (1E, 2E). DI is compared to a liposomal formulation (CTRL).

Formulation	d_0_ (nm)	d_1_ (nm)	DI
CTRL	165 ± 1	90 ± 1	17.75
1	136 ± 1	126 ± 1	109.73
1A	141 ± 1	133 ± 1	142.21
1B	131 ± 3	125 ± 1	176.16
1C	137 ± 1	125 ± 2	92.12
1D	134 ± 1	121 ± 1	83.17
1E	128 ± 2	122 ± 1	172.56
2	140 ± 1	110 ± 2	37.65
2A	137 ± 1	115 ± 1	50.25
2B	126 ± 1	113 ± 1.2	78.20
2C	144 ± 1	112 ± 0.4	36.31
2D	140 ± 2	108 ± 1.1	35.30
2E	132 ± 1	118 ± 0.4	76.08
